# Acute effects of high-potency cannabis flower and cannabis concentrates on everyday life memory and decision making

**DOI:** 10.1038/s41598-021-93198-5

**Published:** 2021-07-02

**Authors:** Carrie Cuttler, Emily M. LaFrance, Amanda Stueber

**Affiliations:** Department of Psychology, Washington State University, PO Box 644820, Pullman, WA 99164-4820 USA

**Keywords:** Neuroscience, Psychology

## Abstract

Statewide legislation has increased public access to high-potency cannabis flower and concentrates, yet federal restrictions limit researchers’ access to relatively low-potency whole-plant cannabis. The goal of this study was to examine the acute effects of high-potency cannabis on cognition using a novel methodology. We further sought to compare cognitive effects of high-potency cannabis flower with and without cannabidiol (CBD), as well as cannabis concentrates to cannabis flower. 80 cannabis users were randomly assigned to stay sober or use their funds to purchase one of three high-potency cannabis products: (1) high-potency flower (≥ 20% THC) without CBD, (2) high-potency flower with CBD, (3) high-potency concentrates (≥ 60% THC) with CBD. Participants were observed over Zoom videoconferencing while inhaling their product or remaining sober and then were administered tests of everyday life memory (prospective, source, temporal order, and false memory) and decision making (risky choice framing, consistency in risk perception, resistance to sunk cost, and over/under confidence) over Zoom. High-potency cannabis flower with CBD impaired free recall, high-potency flower without CBD and concentrates had detrimental effects on source memory, and all three products increased susceptibility to false memories. CBD did not offset impairments and concentrates were self-titrated producing comparable intoxication and impairment as flower.

## Introduction

Changes in statewide legislation have increased public access to a variety of cannabis products in North America, with high-potency cannabis flower (≥ 20% tetrahydrocannabinol [THC]), and extremely high-potency cannabis concentrates (≥ 60% THC) dominating the recreational cannabis market^1^. Nevertheless, because the U.S. Drug Enforcement Administration (DEA) classifies cannabis as a Schedule 1 illicit drug^2^ researchers at federally funded institutions must undergo lengthy approval processes with multiple agencies before they can administer cannabis in their laboratories^3^. Further, approved researchers must use cannabis supplied by the National Institute on Drug Abuse (NIDA), which is presently limited to relatively low potency whole plant cannabis with typical concentrations around 6%^4^. Consequently, our understanding of the acute effects of high-potency cannabis products on humans remains impoverished. Therefore, the goal of the present study was to examine the acute effects of high-potency cannabis on human cognition using a novel methodology which bypasses these legal restrictions.

Previous research has provided robust evidence that verbal free recall and working memory are impaired by the acute administration of cannabis or THC (for reviews see ^5–7^). Further, some limited evidence suggests that cannabidiol (CBD) may offset some of the detrimental effects of THC on memory^8,9^, although a small body of conflicting research suggests that CBD may potentiate effects of THC^10–15^. More specifically, one previous study with humans found that blood levels of THC metabolites were significantly higher following consumption of cannabis containing both THC and CBD, relative to cannabis with equivalent levels of THC, but no CBD^10^. This mirrors the results of several pre-clinical studies demonstrating similar effects in rodents^11–15^.

Nevertheless, effects of cannabis on numerous aspects of everyday life memory have been under-investigated or neglected entirely. More specifically, only three previous studies have investigated acute effects of cannabis on false memory (i.e., recalling events/items which did not occur), with two finding no effects of oral THC on susceptibility to false memories^16,17^. In contrast, a randomized control trial^18^ revealed that vaporized cannabis flower increases false memories for related and unrelated words on the Deese–Roediger–McDermott (DRM) false memory paradigm. Further, only two previous studies have examined acute effects of cannabis on source memory^9,19^, which is the ability to remember the original source of information (e.g., from whom the information was heard or how it was presented) and while neither revealed significant effects, both relied on cannabis containing relatively low levels of THC (< 10%). Finally, no previous research has investigated acute effects of cannabis on temporal order memory (i.e., the ability to remember the sequential order of past events) or prospective memory (i.e., the ability to remember to execute a task in the future, such as taking medication on time).

Similarly, studies examining acute effects of cannabis on decision making have predominantly relied on risky decision-making (e.g., Iowa gambling task) and delay discounting tasks (which assess the propensity to forego larger, delayed rewards for smaller, more immediate ones). Results of studies using such measures of decision making have been equivocal, with authors of two reviews indicating that there are minimal or mixed acute effects of cannabis on decision making^6,20^. Others have suggested that there may be dissociable differences in the effects of cannabis on distinct decision-making functions^5^. However, once again the vast majority of previous research has relied predominantly on risky decision making and delay discounting tasks and to our knowledge, no previous studies have examined the acute effects of cannabis on domains of non-normative decision making such as resistance to sunk cost (i.e., ignoring previous investments when making decisions regarding future circumstances), resistance to framing bias (i.e., the tendency to disregard the framing of problems in terms of possible gains/losses when making decisions), under/overconfidence in one’s own knowledge (i.e., meta-cognition), and consistency in risk perception (i.e., following rules of probability when judging the likelihood of broader vs. narrower risky events). Nevertheless, the results of research on associations between *chronic* cannabis use and decision making have more consistently demonstrated that chronic cannabis users perform worse on decision-making tasks^5,6,20^, with one previous study even revealing evidence that frequency of cannabis use is associated with greater sunk cost propensity^21^.

### Current study

The goal of the current study was to investigate acute effects of high-potency cannabis flower (THC ≥ 20%) and extremely high-potency cannabis concentrates (THC ≥ 60%) on everyday life memory (false, source, temporal order, prospective), and non-normative decision making (resistance to framing bias, consistency in risk perception, under/overconfidence, and resistance to sunk cost) using a novel methodology. Further, this study was designed to address two secondary aims: (1) to compare effects of high-potency cannabis flower with versus without CBD, and (2) to compare effects of extremely high-potency cannabis concentrates to high-potency cannabis flower.

These aims were addressed using a between-subjects field experiment. Specifically, we utilized a novel research strategy, relying on Zoom videoconferencing software to remotely administer cognitive tests to participants who had purchased high-potency cannabis with their own funds and self-administered it in their own homes. Depending on the group to which they had been randomly assigned, participants purchased no product (sober control) or a high-potency cannabis product which fell into one of three categories: (1) THC flower (≥ 20% THC; 0% CBD), (2) THC + CBD flower (≥ 20% THC; ≥ 0.70% CBD), (3) cannabis concentrates (≥ 60% THC; ≥ 0.70% CBD).

## Materials and methods

### Participants

#### Inclusion/exclusion criteria

Participants were required to be fluent in English, 21+ years old, and free of serious neurological and medical conditions (including pregnancy and lactation), learning disabilities, and serious psychiatric disorders. Participants were excluded if they reported heavy alcohol use (> 4 drinks, > 4 times/week), heavy smoking (> 30 cigarettes/week), any illicit drug use in the past 6 months, and/or a history of substance abuse diagnosis or treatment. To reduce risks of adverse reactions, participants had to be experienced cannabis users (i.e., ≥ 50 lifetime uses, current use ≥ 1x/week for ≥ 1/year, experience with both flower and concentrates) and report no serious prior adverse reactions (e.g., panic attacks, psychosis). Participants also had to have access to a computer with a webcam and stable internet connection in a private environment (free of distractions and children) where they could inhale cannabis.

#### Participant characteristics

Results of a power analysis indicate that in order to achieve power of .80 to detect large-sized effects (i.e., $\upeta _{{\text{p}}}^{2}$ = .13) with alpha set at .05, a total sample size of 80 was required. While 86 participants were recruited, participants who did not complete the protocol (*n* = 2) and who purchased a product that did not meet the study criteria (*n* = 4) were excluded. The final sample comprised 80 cannabis users, with 20 assigned to each of the four groups. The sample was well balanced with respect to gender (43.8% women, 56.3% men) and ranged in age from 21 to 44 (*M* = 23.87; *SD* = 5.67). The average age of onset of cannabis use was 17.01 (*SD* = 1.98), and participants had used cannabis for an average of 5.66 (*SD* = 5.30) years. There were no significant differences in any demographic characteristics or cannabis use patterns across the four groups suggesting random assignment was successful in producing groups equivalent at baseline (see Table 1).

**Table 1:** Demographic characteristics and cannabis use patterns across groups.

	Sober *n* = 20	THC *n* = 20	THC + CBD *n* = 20	Concentrate *n* = 20	
**Demographics**	**%**	**%**	**%**	**%**	
% Female	50%	45%	30%	50%	$\chi$^2^(3) = 2.18, *p* = .54, Φ = .17
% White	65%	80%	85%	85%	$\chi$^2^(3) = 3.21, *p* = .36, Φ = .20

	*M* (*SD*)	*M* (*SD*)	*M* (*SD*)	*M* (*SD*)	
Age	25.25 (7.37)	23.50 (5.61)	22.75 (4.66)	24.00 (4.77)	*F*(3,76) = 0.68, *p* = .57, $\upeta _{{\text{p}}}^{2}$ ﻿= .03
BMI	24.65 (4.14)	24.76 (5.19)	25.73 (4.95)	25.24 (5.19)	*F*(3,76) = 0.21, *p* = .89, $\upeta _{{\text{p}}}^{2}$ = .01
Verbal IQ (WTAR)	38.90 (67.26)	40.65 (5.01)	37.80 (6.28)	37.45 (6.69)	*F*(3,76) = 1.03, *p* = .38, $\upeta _{{\text{p}}}^{2}$ = .04

Cannabis use patterns	*M* (*SD*)	*M* (*SD*)	*M* (*SD*)	*M* (*SD*)	
Cannabis abstinence (h)	21.72 (11.30)	28.60 (22.71)	23.72 (20.09)	19.38 (6.33)	*F*(3,76) = 1.13, *p* = .34, $\upeta _{{\text{p}}}^{2}$ = .04
Frequency^a^	7.10 (1.41)	6.95 (1.61)	7.35 (1.35)	6.75 (2.05)	*F*(3,76) = 0.48, *p* = .69, $\upeta _{{\text{p}}}^{2}$ = .02
Cannabis quantity^b^	4.10 (0.97)	3.90 (0.91)	4.10 (0.91)	3.75 (0.91)	*F*(3,76) = 0.68, *p* = .57, $\upeta _{{\text{p}}}^{2}$ = .03
Years cannabis use	8.20 (7.74)	4.37 (3.52)	4.60 (4.20)	5.45 (4.14)	*F*(3,76) = 2.31, *p* = .08, $\upeta _{{\text{p}}}^{2}$ = .08
Age of onset of cannabis use	16.45 (2.04)	17.75 (1.65)	17.15 (1.53)	16.70 (2.45)	*F*(3,76) = 1.71, *p* = .17, $\upeta _{{\text{p}}}^{2}$ = .06

*BMI* body mass index, *WTAR* Weschler’s Test of Adult Reading.

^a^Participants rated the number of days of the past month on which they used cannabis using the following scale: 1 = 1–3 days, 2 = 4 – 6 days, 3 = 7–10 days, 4 = 11–14 days, 5 = 15–19 days, 6 = 19–22 days, 7 = 23–26 days, 8 = 27–31 days.

^b^Participants rated the average number of puffs they typically took during cannabis use sessions on a scale where 1 = 0–2 puffs, 2 = 3–5 puffs, 3 = 6–8 puffs, 4 = 9–11 puffs, and 5 = 12 or more puffs.

### Materials

#### Weschler test of adult reading (WTAR)

The WTAR is a brief measure of premorbid verbal IQ^22^. The test consists of a list of 50 words that participants pronounce aloud. They are given 1 point for each correctly pronounced word. This test was used to ensure groups had comparable cognitive abilities at baseline.

#### Prospective memory tests

Participants’ ability to remember to execute tasks in the future was assessed with two prospective memory tests: the reminder test^23–25^ and difficulty ratings test^26^. For the difficulties ratings test participants were asked to rate the difficulty of each test they completed immediately after completing each, using a 0 to 10 scale. The percentage of tests each participant provided difficulty ratings for was computed. For the more motivationally salient reminder test, participants were required to remind the experimenter to email them their $40 Amazon e-gift card at the end of the study, during the debriefing. The reminder test was scored in a binary manner (0 = no reminder at appropriate time, 1 = reminder).

#### Source memory test

For the source memory test^26^, participants saw 32 basic pictures (e.g., black and white line drawings of common objects and animals such as an envelope, hat and horse) and printed words for 2-s each. After a 10-min retention interval, they freely recalled as many items as possible. Next, they were presented with 64 words and were asked to identify whether each was presented previously as a picture or a word or whether the word was not presented earlier. Source memory discrimination indices were computed based on the single source conditional-source identification measure^26,27^. Specifically, the discrimination index (DI) for picture memory was calculated as [Correct_pic_/(Correct_pic_ + False Alarms_pic_)], and the DI for word memory was calculated as [Correct_word_/(Correct_word_ + False Alarms_word_)] where Correct represents the number of pictures/words that were correctly identified, and False Alarms represent the number of pictures/words that were incorrectly identified. Therefore, the DIs for picture and word memory represent the proportion of total pictures/words that were accurately identified.

#### Deese–Roediger–McDermott false memory paradigm (DRM)

During the DRM^28^, participants heard six lists of 12 words that each related to one critical lure word which was not on the list. For example, a list might include the words: tired, pillow, bed, dream, and night, while the critical lure word ‘sleep’ that directly relates to each of the words in the list is never presented. For the free recall trial, participants recalled as many words as they could from each list immediately after hearing each. After a 10-min retention interval, participants were presented with six new lists of seven words which contained two old words, one critical lure, two new related words (i.e., words which were related to the theme of an original list, but not as directly related as the critical lure word), and two new unrelated words (i.e., words that are completely unrelated to the theme of the original list). False identification of critical lures, related words, and unrelated words was used to measure false memory.

#### Temporal order memory test

To assess memory for the sequential order of previous events^26^, participants were asked to freely recall all the tasks they completed during the testing session, in the order in which they were completed. The total number of tests they correctly recalled was scored as a measure of free recall. Participants’ responses were then individually scored from 0 to 2. Participants received a score of 2 for each task they recalled in the correct sequence. If they switched the order of a task with an adjacent task or recalled a series of tests in the correct order, but in the incorrect location within the larger list, they received a score of 1 for each test. If participants recalled tests in an order that was completely out of sequence, they received a score of 0 for each test. Individual scores were then summed to create a total temporal order memory recall score. Finally, participants were presented with 10 pairs of tasks that they completed and were asked to indicate which of the two tasks they completed first. Participants received a total temporal order recognition score which reflected the number of questions they answered correctly.

#### Under/overconfidence test

For the under/overconfidence test of meta-cognition (i.e., participants’ awareness of their own knowledge)^29^, participants were presented with 15 true/false statements (e.g., True or False: Amman is the capital of Jordan), and were asked to indicate whether each statement is true or false and to rate how confident they were in that decision, using a scale ranging from 50% (just guessing), to 100% (absolutely sure). The percentage of true/false questions that were correctly answered was subtracted from the average confidence rating. As such, negative scores reflect under-confidence, and positive scores reflect over-confidence.

#### Resistance to framing test

The resistance to framing test^29^ examines whether decisions are impacted by the manner in which problems are framed. The test is divided into two parts; a gain framing trial and a loss framing trial, the order of which were counterbalanced. For the gain framing trial, participants were presented with seven scenarios with two response options framed in terms of gains. For example, in a gain-framing trial, participants would be presented with hypothetical scenarios, such as: “Imagine that the U.S. is preparing for the outbreak of an unusual disease, which is expected to kill 600 people. Two alternative programs to combat the disease have been proposed. Assume that the exact scientific estimates of the consequences of the programs are as follows: If Program A is adopted, 200 people will be saved. If Program B is adopted, there is a 33% chance that 600 people will be saved, and a 67% chance that no people will be saved.” For the loss-framing trial, participants were presented with the same seven scenarios; however, the two response options were framed in terms of losses. For example, participants were presented with the same scenario, but this time were informed that: “If Program A is adopted, 400 people will die. If Program B is adopted, there is a 33% chance that nobody will die, and a 67% chance that 600 people will die. On both trials, participants were asked to indicate their preference between the two options using a 6-point scale. The absolute difference in responses between paired questions was computed and averaged. Lower scores indicate higher resistance to framing.

#### Consistency in risk perception test

The consistency in risk perception test^29^ measures participants’ ability to follow rules of probability while judging the likelihood of broader versus narrower risky events. Participants rated the probability of 10 different events occurring in the next 5 years. Events were paired, with one of each pair being broad in nature (e.g., the probability that someone will steal from you in the next 5 years), and the other being narrower in focus (e.g., the probability that someone will break into your home and steal something from you in the next 5 years). The number of times participants appropriately judged the broader event to be more likely than its more narrowly focused pair was scored, with consistent judgements receiving a score of 2 points per pair.

#### Resistance to sunk cost test

The resistance to sunk cost test^29^ assesses participants’ tendency to ignore previous investments when making decisions regarding future circumstances. Participants were presented with a series of scenarios and were asked to rate the likelihood that they would select either (1) a response option that is less desirable, but preserves a previous investment, or (2) a response option that is more desirable but involves a loss of a previous investment. For example, “You are in a hotel for one night and paid $6.95 to watch a movie on pay-per-view TV. You then discover there is a movie you would much rather see on one of the free cable channels. Would you be more likely to watch the movie you paid for, or the one on the free cable channel?” Participants were asked to indicate their preference between the two options on a 6-point scale [1 = absolutely prefer the option that preserves a previous investment (e.g., watching the movie you paid for), 6 = absolutely prefer the option that involves a loss of a previous investment (e.g., watching the free movie)]. Scores were averaged with higher scores indicating higher resistance to sunk cost.

### Procedures

**Figure 1:**
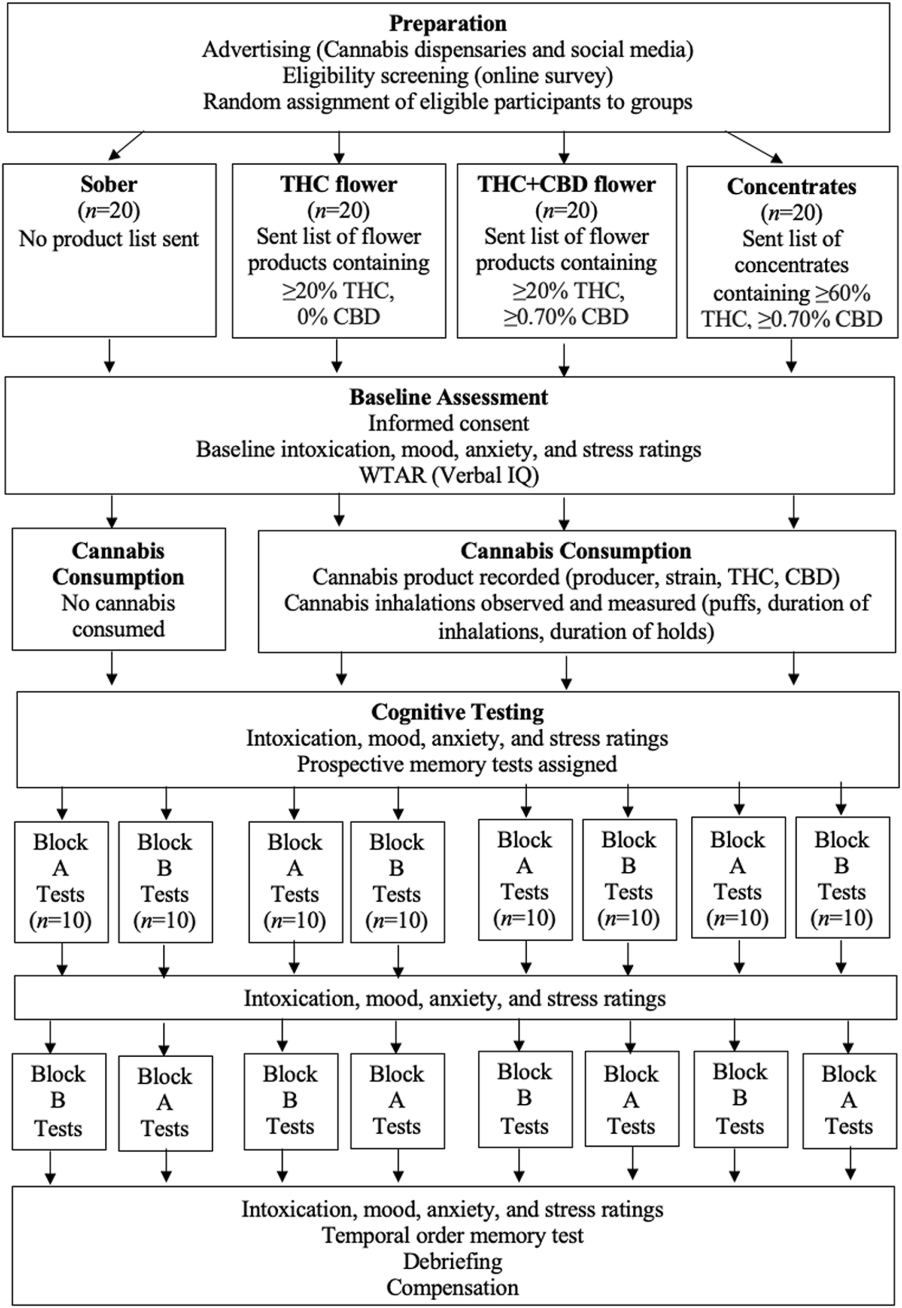
Overview of study procedures. Block A contained the source memory test, the under/overconfidence test of decision making, and the first half of the resistance to framing test (gains framing trials). Block B contained the DRM false memory paradigm, the consistency in risk perception test, and the other half of the resistance to framing test (loss framing trials). Block A and B were completely counterbalanced with 10 participants per group per order in order to control for order effects (e.g., fatigue, strength of drug effects).

Figure 1 displays an overview of the study procedures.

#### Preparation

The study was reviewed by our university’s Office of the Attorney General and was approved by the Washington State University Institutional Review Board. The research was conducted in accordance with the Declaration of Helsinki. Participants were recruited through advertisements posted online (e.g., Facebook, Craigslist) as well as via printed flyers in cannabis dispensaries in Washington state where recreational cannabis is legal. Prospective participants completed an online survey to determine their eligibility, obtain basic demographic and cannabis consumption information. Eligible participants were randomly assigned to one of the four groups and were emailed a Zoom link for their testing session. Those in the three cannabis-using groups were informed that we are interested in studying the effects of specific products and were sent a list of products available at local recreational cannabis dispensaries that met criteria for the group to which they were assigned (e.g., participants randomly assigned to the THC flower group were emailed a list of pre-rolled joints with ≥ 20% THC and 0% CBD available at local dispensaries). Prior to testing, participants in the cannabis-using groups purchased a product off the list using their own funds. All participants were required to abstain from using any cannabis products for a minimum of 8 h prior to testing and those in the sober control group were further required to remain sober during the testing session.

#### Baseline assessment

After initiating the Zoom session, the researcher obtained informed consent online. Participants then indicated how many hours ago they last used cannabis and provided baseline ratings of intoxication, anxiety, mood, and stress using 0 (not at all) to 10 (extremely) rating scales. To ensure random assignment produced groups equivalent in cognitive ability at baseline, participants also completed the WTAR while sober.

#### Cannabis consumption

Participants in the three cannabis-using groups were asked to show the researcher the cannabis product they purchased for the study. The brand, strain, and cannabinoid content (%THC and %CBD) were recorded. The vast majority of participants used the list they were sent to purchase the product-type to which they had been randomly assigned to use. As described previously, the small number (*n* = 4) who purchased a product that did not meet the study criteria were excluded from data analyses.

Participants were shown the paced puff procedure^30^ using screen sharing and were observed while they inhaled their cannabis product. The researcher recorded the number of puffs and duration of inhalations and holds until the participant self-titrated but did not enforce adherence to the paced puff procedure, due to federal legal restrictions.

#### Cognitive testing

Participants again rated their intoxication, mood, anxiety, and stress using the same 0 to 10 scales. Then, they received instructions for the prospective memory tests. The majority of the remaining tests were divided into two testing blocks (A and B) that were counterbalanced to control for potential order effects. Ten people in each of the four groups completed each order to achieve perfect counterbalancing. Block A began with the source memory test. During the retention interval for this test, participants completed the under/overconfidence test of decision making, and the first half of the resistance to framing test (gain framing trials). At the end of Block A (which required ~ 25 min), participants again rated their intoxication, mood, anxiety, and stress. Block B began with the DRM false memory paradigm. During the retention interval for this test, participants completed the consistency in risk perception test and the other half of the resistance to framing test (loss framing trials). Next, they completed the resistance to sunk cost measure. At the end of Block B (which required ~ 25 min), participants re-rated their intoxication, mood, anxiety, and stress. After both testing blocks were completed, the temporal order memory test was administered. Finally, participants were debriefed and emailed their compensation in the form of a $40 Amazon e-gift card. For all tests, verbal instructions were provided over Zoom along with written instructions presented via the screen sharing option in Zoom. Screen sharing was also used to display word lists, pictures and other stimuli.

### Data analysis

Data were screened for univariate outliers defined as scores > 3.29 standard deviations from the mean^31^. Eight outliers (< 0.5% of the data) were detected and trimmed to one unit higher/lower than the nearest non-outlying value^31^. The reminder prospective memory test was scored in a binary manner and analyzed using chi-square. One-way analyses of variance (ANOVAs) and a priori planned pairwise LSD comparisons were used to compare the four groups’ performance on the remaining cognitive tests. Mixed factorial ANOVAs were used to compare changes in intoxication over time across the groups. IBM SPSS (version 26) was used to conduct all analyses, with pairwise deletion used for missing data (1% of the total dataset).

## Results

### Cannabis consumption and intoxication ratings

**Figure 2:**
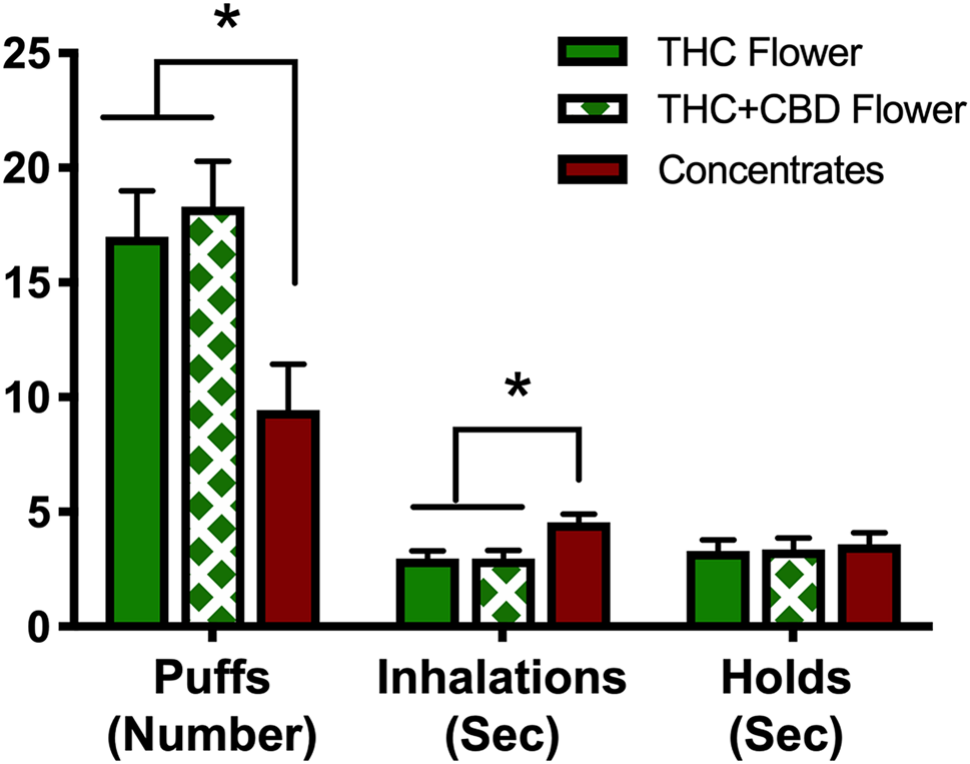
Cannabis consumption across groups. Bars represent mean number of puffs, mean duration of inhalations, and mean durations of holds in the THC flower, THC + CBD flower, and concentrates groups. Error bars represent standard errors of the mean. The three groups showed significant differences in number of puffs across the three cannabis-using groups, *F*(2,57) = 5.79, *p* = .005, $\upeta _{{\text{p}}}^{2}$ ﻿= .17. Follow up post hoc comparisons indicated that the concentrates group took significantly fewer puffs than the THC flower (*p* = .009) and THC + CBD flower (*p* = .003) groups. The difference between the two flower groups was not significant (*p* = .65). The three groups also differed with respect to mean duration of inhalations, *F*(2,57) = 7.03, *p* = .002, $\upeta _{{\text{p}}}^{2}$ ﻿= .20. Follow-up comparisons indicated that the concentrates group took significantly longer inhalations than the two flower groups (*p*s = .002) who did not significantly differ from one another (*p* = .99). There were no significant differences in the mean duration of holds across the three groups, *F*(2,56) = 0.11, *p* = .90, $\upeta _{{\text{p}}}^{2}$ = .004.

Average THC concentrations were 22.82% (*SD* = 3.31) in the THC flower group, 22.81% (*SD* = 1.58) in the THC + CBD flower group, and 71.43% (*SD* = 15.88) in the concentrates group. Average CBD concentrations were 0.00% (*SD* = 0.00) in the THC flower group, 1.32% (*SD* = 0.51) in the THC + CBD flower group, and 2.20% (*SD* = 1.60) in the concentrates group. Figure 2 displays cannabis consumption information broken down by group. The results show that participants in the concentrate group took significantly fewer puffs and had significantly longer mean inhalation durations. There were no group differences in duration of holds.

**Figure 3:**
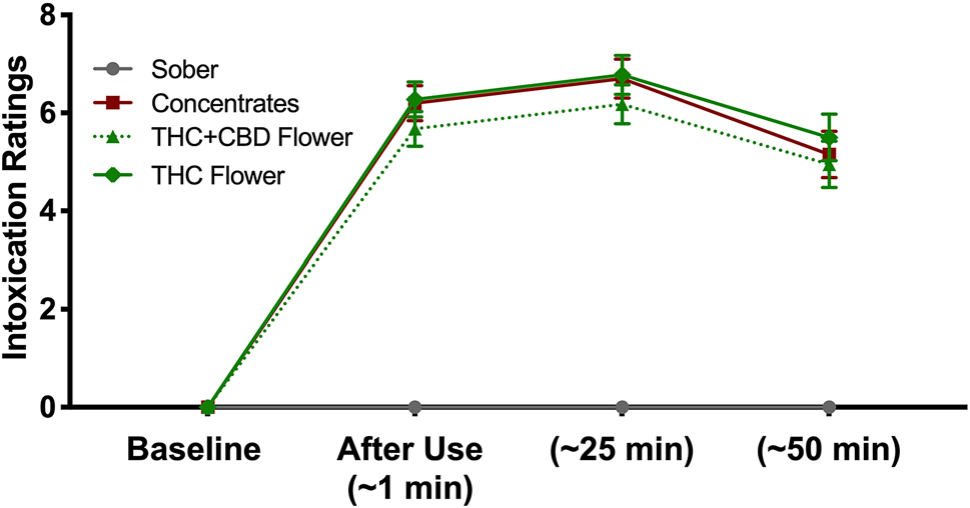
Intoxication ratings across groups over time. Lines represent mean intoxication ratings before, immediately after, 25 min after, and 50 min after inhaling cannabis in the four groups. Error bars represent standard errors of the mean. Results revealed significant main effects of time, *F*(3,228) = 261.59, *p* < .001, $\upeta _{{\text{p}}}^{2}$ = .77, and group, *F*(3,76) = 73.15, *p* < .001, $\upeta _{{\text{p}}}^{2}$ = .74, as well as a significant time x group interaction, *F*(9,228) = 29.32, *p* < .001, $\upeta _{{\text{p}}}^{2}$ = .54. Follow-up one-way within-groups ANOVAs revealed no change in intoxication ratings over time in the sober control group (they consistently remained at 0). In contrast there were significant changes in intoxication ratings in the THC flower group, *F*(3,57) = 106.52, *p* < .001, $\upeta _{{\text{p}}}^{2}$ = .85, THC + CBD flower group, *F*(3,57) = 73.27, *p* < .001, $\upeta _{{\text{p}}}^{2}$ = .79, and concentrates group, *F*(3,57) = 85.51, *p* < .001, $\upeta _{{\text{p}}}^{2}$ = .82. Post hoc comparisons revealed that for all three cannabis-using groups intoxication ratings significantly increased from before cannabis use to all three time points after use (*p*s < .001). There were also small but significant decreases in these ratings from 25 to 50 min after use (*p*s ≤ .001) in the three cannabis-using groups. Intoxication ratings at baseline were 0 for all participants in all groups. There were significant differences in the four groups’ intoxication ratings 1 min after the cannabis inhalation session, *F*(3, 76) = 73.36, *p* < .001, $\upeta _{{\text{p}}}^{2}$ = .74, 25 min after, *F*(3, 76) = 68.36, *p* < .001, $\upeta _{{\text{p}}}^{2}$ = .73, and 50 min after use, *F*(3, 76) = 30.41, *p* < .001, $\upeta _{{\text{p}}}^{2}$ = .55. Further post hoc comparisons revealed that all three cannabis-using groups had significantly higher intoxication ratings than the sober control group at all three time points (*p*s < .001). There were no significant differences in the intoxication ratings of any of the cannabis-using groups at any time point (*p*s > .24).

Figure 3 displays each group’s mean intoxication ratings over time. There was a significant increase in intoxication ratings from baseline to 1 min, 25 min, and 50 min after use in the three cannabis groups (*p*s < 0.001). There were no significant differences in the intoxication ratings of the three cannabis-using group at any time point.

### Mood, anxiety, and stress ratings

**Figure 4:**
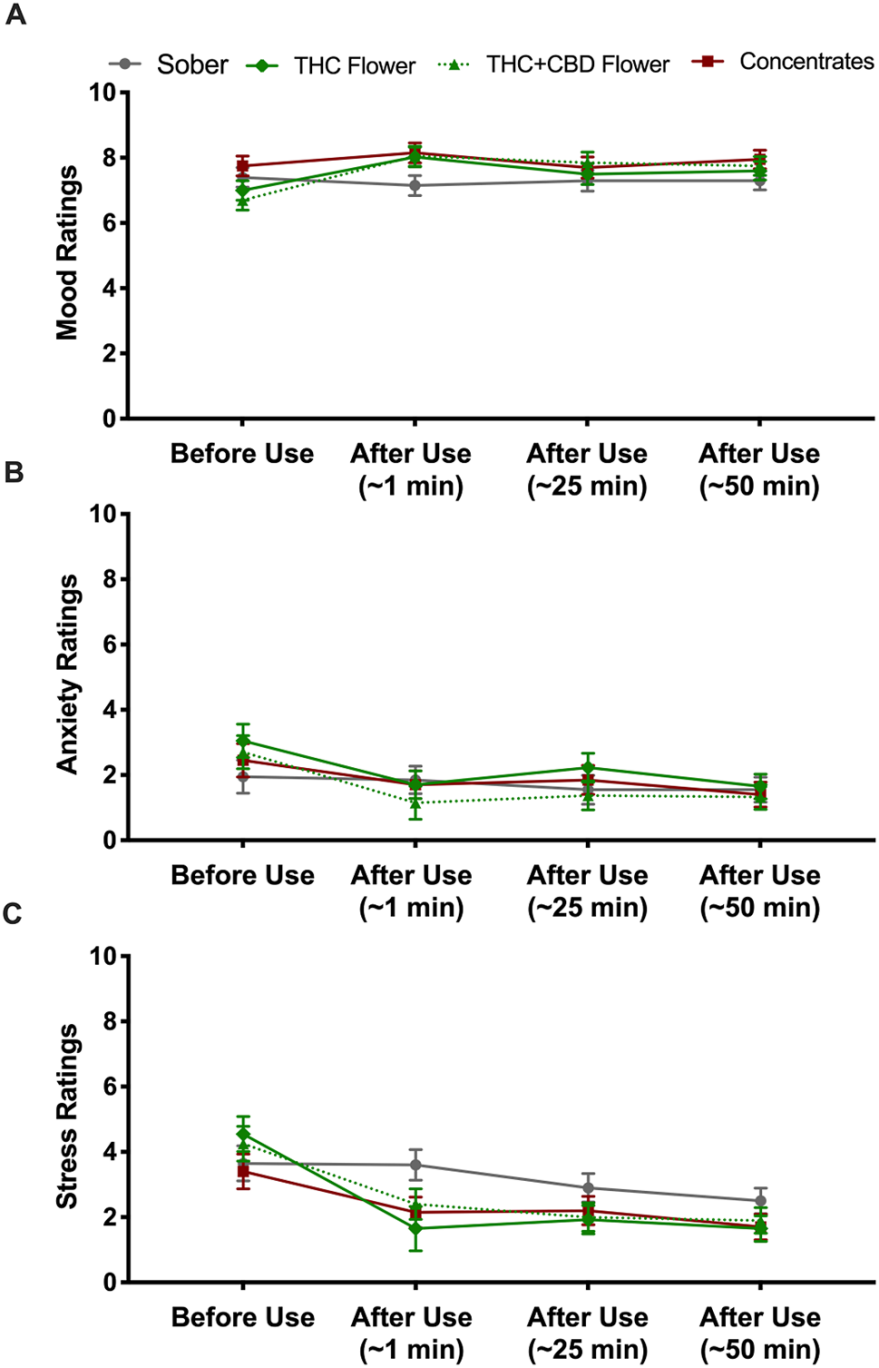
Changes in Mood (**A**), Anxiety (**B**), and Stress (**C**) over the Course of the Study. (**A**) Lines represent mean mood ratings before, immediately after, 25 min after, and 50 min after the cannabis inhalation session in the four groups. Ratings were made on a 0 to 10 scale with 0 representing extremely negative and 10 representing extremely positive. Error bars represent standard errors of the mean. Results revealed significant main effects of time, *F* = 6.12, *p* = .001, $\upeta _{{\text{p}}}^{2}$ ﻿= .07, but not group, *F* = 1.05, *p* = .37, $\upeta _{{\text{p}}}^{2}$ = .04. These effects were qualified by a significant time x group interaction, *F* = 2.31, *p* = .02, $\upeta _{{\text{p}}}^{2}$ = .08. Follow-up one-way within-groups ANOVAs revealed no significant changes in mood over time in the sober group, *F* = 0.35, *p* = .80, $\upeta _{{\text{p}}}^{2}$ = .02, or the concentrates group, *F* = 0.95, *p* = .42, $\upeta _{{\text{p}}}^{2}$ = .05. However, there were changes in mood over time in the THC flower group, *F* = 4.00, *p* = .01, $\upeta _{{\text{p}}}^{2}$ = .17, the THC + CBD flower group, *F* = 5.89, *p* = .001, $\upeta _{{\text{p}}}^{2}$ = .24. Post hoc comparisons further revealed an increase in mood from before to 1 min after using cannabis in the THC flower group. Contrasts of the other time points revealed no significant differences in this group. The THC + CBD flower group reported significant elevations in mood from before to 1 min (*p* = .002), 25 min (*p* = .002), and 45 min (*p* = .047) after using cannabis. No other contrasts involving time were significant. Additional one-way between-groups ANOVAs revealed no overall group differences in mood ratings at baseline, *F* = 2.34, *p* = .08, $\upeta _{{\text{p}}}^{2}$ = .08, 1 min after use, *F* = 2.35, *p* = .08, $\upeta _{{\text{p}}}^{2}$ = .08, 25 min after use, *F* = 0.55, *p* = .65, $\upeta _{{\text{p}}}^{2}$ = .02, or 50 min after use, *F* = 0.92, *p* = .43, $\upeta _{{\text{p}}}^{2}$ = .04. (**B**) Lines represent mean anxiety ratings before, immediately after, 25 min after, and 50 min after the cannabis inhalation session in the four groups. Ratings were made on a 0 to 10 scale with 0 representing not at all anxious and 10 representing extremely anxious. Error bars represent standard errors of the mean. Results revealed significant main effects of time, *F* = 15.60, *p* < .001, $\upeta _{{\text{p}}}^{2}$ = .17, but not group, *F* = 0.34, *p* = .80, $\upeta _{{\text{p}}}^{2}$ = .01. The interaction between time x group was not statistically significant, *F* = 1.51, *p* = .15, $\upeta _{{\text{p}}}^{2}$ = .06. Post hoc comparisons further revealed that across all four groups combined anxiety ratings decreased from before cannabis use to 1 min (*p* < .001), 25 min (*p* = .001) and 50 min (*p* < .001) after the cannabis use session. There was also a small but significant decrease in anxiety from 25 to 50 min after use (*p* = .006). No other contrasts involving time were statistically significant. Finally, one-way between-groups ANOVAs confirmed there were no overall group differences in anxiety ratings at baseline, *F* = 0.83, *p* = .48, $\upeta _{{\text{p}}}^{2}$ = .03, 1 min after use, *F* = 0.58, *p* = .66, $\upeta _{{\text{p}}}^{2}$ = .02, 25 min after use, *F* = 0.71, *p* = .55, $\upeta _{{\text{p}}}^{2}$ = .03, or 50 min after use, *F* = 0.15, *p* = .93, $\upeta _{{\text{p}}}^{2}$ = .006. (**C**) Lines represent mean stress ratings before, immediately after, 25 min after, and 50 min after inhaling cannabis in the four groups. Ratings were made on a 0 to 10 scale with 0 representing not at all stressed and 10 representing extremely stressed. Error bars represent standard errors of the mean. Results revealed significant main effects of time, *F* = 38.58, *p* < .001, $\upeta _{{\text{p}}}^{2}$ = .34, but not group, *F* = 0.87, *p* = .46, $\upeta _{{\text{p}}}^{2}$ = .03. These were qualified by a significant time x group interaction, *F* = 3.17, *p* = .001, $\upeta _{{\text{p}}}^{2}$ = .11. Follow-up one-way within-groups ANOVAs revealed significant changes in stress over time in the sober group, *F* = 12.71, *p* < .001, $\upeta _{{\text{p}}}^{2}$ = .40. Post hoc comparisons showed significant decreases in stress ratings in the sober group from the first to third (*p* = .004) and fourth (*p* = .001) time points as well as further decreases from time point three to four (*p* = .03). The difference between the first and second time points was null (*p* = .33). There were also significant changes in stress in the THC + CBD flower group, *F* = 18.49, *p* < .001, $\upeta _{{\text{p}}}^{2}$ = .49. Post hoc comparisons showed significant decreases in stress ratings from the first to second (*p* < .001), third (*p* = .001) and fourth (*p* < .001) time points only. Similarly, there were significant changes in stress in the THC flower group, *F* = 10.14, *p* < .001, $\upeta _{{\text{p}}}^{2}$ = .35. Post hoc comparisons showed significant decreases in stress ratings from the first to second (*p* = .002), third (*p* = .001) and fourth (*p* < .001) time points only. There were also significant changes in stress in the concentrate group, *F* = 6.25, *p* = .001, $\upeta _{{\text{p}}}^{2}$ = .25. Post hoc comparisons showed significant decreases in stress ratings from the first to second (*p* = .005), third (*p* = .045) and fourth (*p* < .001) time points only. Finally, additional one-way between-groups ANOVAs revealed no overall group differences in stress ratings at baseline, *F* = 0.99, *p* = .40, $\upeta _{{\text{p}}}^{2}$ = .04, 25 min after use, *F* = 1.05, *p* = .38, $\upeta _{{\text{p}}}^{2}$ = .04, or 50 min after use, *F* = 0.99, *p* = .40, $\upeta _{{\text{p}}}^{2}$ = .04. However, there were significant differences across groups 1 min after use, *F* = 3.13, *p* = .03, $\upeta _{{\text{p}}}^{2}$ = .11. Further post hoc comparisons indicated that the THC flower and concentrates groups had significantly lower stress ratings than the sober control group (*p* = .004 and *p* = .03 respectively) immediately after use.

Figure 4 displays changes in mood (A), anxiety (B), and stress (C) over time for the four groups. As further detailed in the figure caption, anxiety and stress generally decreased over time with subtle differences in these changes across groups. Importantly, there were no group differences in ratings of mood, anxiety, or stress at any given time point, with the exception of stress ratings 1 min after use which were significantly lower in the THC flower and concentrates groups than the sober control group. ANCOVAs confirmed these differences in stress ratings did not account for any group differences in the cognitive test performance reported below.

### Cognitive test performance

Table 2 displays each group’s performance on the cognitive tests, results of the one-way ANOVAs, and planned post hoc comparisons. Standard (unadjusted) *p* values are presented in the table along with Benjamini–Hochberg adjusted *p* values^32^ that control for false discovery rate. As shown in the table, significant effects of high-potency cannabis were detected only on the source and false memory tests. Note that the effect on picture recall was significant at the conventional 0.05 level but did not survive the Benjamini–Hochberg adjustment for multiple comparisons. Nevertheless, results of a priori planned comparisons indicated that the THC + CBD flower group had significantly poorer free recall of pictures on the source memory test than the sober control group (*p* = .01), THC flower group (*p* = .02), and the concentrates group (*p* = .03) (see Table 2). Similarly, the THC + CBD flower group performed significantly worse on the free recall trial of the false memory test (*p* = .02). These results indicate that high-potency cannabis with CBD is detrimental to free recall. Further, as shown in the table, the THC flower and concentrates groups had lower discrimination indices for pictures on the source memory test relative to the sober control group (*p*s < .001) (see Table 2). Finally, relative to the sober control group the THC flower group (*p* = .006), THC + CBD flower group (*p* < .001) and concentrates group (*p* < .001) demonstrated an increased susceptibility to false memories for unrelated words, while the concentrates group further showed increased susceptibility to false memories for related words (*p* = .04) (see Table 2).

**Table 2:** Cognitive test performance.

	Sober *n* = 20 M (*SD*)	THC *n* = 20 ﻿M (*SD*)	THC + CBD *n* = 20 ﻿M (*SD*)	Concentrate *n* = 20 ﻿M (*SD*)	Inferential statistics	Benjamini–Hochberg adjusted *p*	Effect sizes
**Prospective memory tests**							
Reminder test	90%	85%	75%	90%	$\chi$ ^2^(3) ﻿= 2.35, *p* = .50	.77	Φ = .17
Difficulty ratings test	56.88% (27.20)	47.50% (31.44)	57.81% (26.74)	49.06% (31.70)	*F*(3,76) = 0.65, *p* = .59	.77	$\upeta _{{\text{p}}}^{2}$ ﻿= .02
**Source memory test**							
Total free recall pictures	4.65 (1.90)^a^	4.53 (2.20)^a^	3.05 (2.11)^b^	4.45 (1.76)^a^	*F*(3,75) = 2.81, *p* = .04	.23	$\upeta _{{\text{p}}}^{2}$ = .10
Total free recall words	1.60 (1.14)	1.16 (1.01)	1.65 (1.79)	1.50 (1.32)	*F*(3,75) = 0.52, *p* = .67	.81	$\upeta _{{\text{p}}}^{2}$ = .02
Source memory DI pictures	0.92 (0.09)^a^	0.73 (0.23)^b^	0.82 (0.14)^ab^	0.73 (0.15)^b^	*F*(3,75) = 5.89, *p* = .001	.009	$\upeta _{{\text{p}}}^{2}$ = .19
Source memory DI words	0.62 (0.19)	0.52 (0.23)	0.52 (0.17)	0.59 (0.20)	*F*(3,75) = 1.16, *p* = .33	.80	$\upeta _{{\text{p}}}^{2}$ = .04
**False memory test**							
Total free recall	32.26 (7.19)^a^	30.40 (7.78)^ab^	27.05 (7.00)^b^	29.10 (5.88)^ab^	*F*(3,75) = 1.92, *p* = .13	.55	$\upeta _{{\text{p}}}^{2}$ = .07
False memory: critical lures	4.84 (0.90)	4.75 (1.48)	4.80 (1.36)	5.10 (0.97)	*F*(3,75) = 0.33, *p* = .80	.85	$\upeta _{{\text{p}}}^{2}$ = .01
False memory: related	4.37 (2.36)^a^	5.55 (1.89)^ab^	5.35 (2.13)^ab^	5.85 (2.52)^b^	*F*(3,75) = 1.59, *p* = .20	.68	$\upeta _{{\text{p}}}^{2}$ = .06
False memory: unrelated	0.68 (0.82)^a^	2.45 (2.72)^b^	3.25 (2.17)^b^	2.45 (2.72)^b^	*F*(3,75) = 6.05, *p* < .001	.02	$\upeta _{{\text{p}}}^{2}$ = .19
**Temporal order memory test**							
Total free recall	8.75 (3.06)	8.45 (4.00)	6.80 (2.57)	7.45 (3.38)	*F*(3,76) = 1.49, *p* = .22	.62	$\upeta _{{\text{p}}}^{2}$ = .06
Temporal order recall	4.30 (3.50)	3.60 (4.28)	2.90 (3.19)	3.05 (2.80)	*F*(3,76) = 0.66, *p* = .58	.82	$\upeta _{{\text{p}}}^{2}$ = .03
Temporal order recognition	7.70 (1.34)	7.50 (1.79)	7.80 (1.15)	7.50 (1.28)	*F*(3,76) = 0.23, *p* = .88	.88	$\upeta _{{\text{p}}}^{2}$ = .01
**Decision making tests**							
Over/under confidence	− 8.12 (7.62)	− 8.46 (5.83)	− 5.44 (5.43)	− 8.87 (8.15)	*F*(3,76) = 1.02, *p* = .39	.74	$\upeta _{{\text{p}}}^{2}$ = .04
Risk perception	6.30 (2.08)	7.60 (5.53)	6.50 (2.24)	5.80 (2.04)	*F*(3,76) = 1.05, *p* = .38	.81	$\upeta _{{\text{p}}}^{2}$ = .04
Framing bias	1.19 (0.51)	1.05 (0.72)	1.27 (0.78)	1.21 (0.76)	*F*(3,76) = 0.37, *p* = .78	.88	$\upeta _{{\text{p}}}^{2}$ = .01
Sunk cost bias	4.44 (0.97)	4.28 (0.68)	4.08 (0.58)	4.26 (0.56)	*F*(3,76) = 0.90, *p* = .45	.76	$\upeta _{{\text{p}}}^{2}$ = .03

Values in Reminder Test row reflect the % of participants in each group who succeeded on the test while those in the Difficulties Rating Test row reflect the mean % of tests for which a difficulty rating was provided. Means with no superscripts or shared superscripts do not differ significantly while those with different superscripts are significantly different at *p* < .05. $\upeta _{{\text{p}}}^{2}$ = .01 is small, $\upeta _{{\text{p}}}^{2}$ = .06 is medium, $\upeta _{{\text{p}}}^{2}$ = .14 is large.

## Discussion

The goal of this study was to examine acute effects of high-potency cannabis flower (≥ 20% THC) and concentrates (≥ 60%) on everyday life memory and decision making using a novel methodology that bypasses DEA prohibitions, including those that require researchers to utilize relatively low-potency (~ 6% THC) whole-plant cannabis from the NIDA drug supply. Consistent with previous research relying predominantly on relatively low-potency cannabis, results from the present study generally show that cannabis acutely impairs free recall (for reviews see^5–7^) and increases susceptibility to false memories for related and unrelated words but not critical lures^18^. Moreover, to our knowledge, this is the first study to demonstrate acute detrimental effects of cannabis on source memory.

While the goal of this study was to examine the acute effects of high-potency cannabis on cognition, perhaps one of the most interesting and novel findings was that participants randomly assigned to use a cannabis concentrate self-titrated after significantly fewer puffs yet reported comparable levels of intoxication and demonstrated equivalent levels of impairment as those who inhaled the flower products. There has been concern and speculation that extremely high-potency cannabis concentrates will magnify harms, but the absence of cannabis concentrates in the NIDA drug supply have resulted in very limited research on their actual use or effects on humans. Present findings indicate that experienced cannabis users simply use less of these higher potency products to achieve the same levels of intoxication and impairment. As such, it is possible that concentrates may even reduce harm by diminishing the amount of the product that is inhaled into the lungs. Clearly future research is needed to better understand whether concentrates enhance or mitigate the potentially detrimental effects of acute cannabis use on physical health and/or cognition.

This is the first study to examine acute effects of cannabis on prospective memory, temporal order memory, resistance to framing and sunk cost biases, under/overconfidence, and consistency in risk perception. Despite the use of high-potency products, we failed to detect any significant effects on any of these outcomes. It is possible that this reflects a true absence of effects of cannabis on these aspects of cognition. Indeed, the null findings on tests of non-normative decision making are consistent with the conclusions of some previous literature which has demonstrated minimal or mixed acute effects of cannabis on decision making^5,6,20^. For instance, Broyd et al.^5^ reported evidence that acute THC administration may increase risk taking and alter sensitivity to reward and punishment, though not all studies included in the review provided evidence for acute cannabis-induced impairments in decision making and it is unclear in what contexts or conditions these findings are detected. In general, it appears that the acute impacts of cannabis on decision making processes are far less reliable and robust than its effects on free recall.

It is also possible that we failed to detect significant differences on the measures of prospective memory, temporal order memory, and non-normative decision making because participants in all four groups were already impaired by the chronic use of cannabis and that the acute effects of cannabis simply do not extend beyond the chronic effects. While to our knowledge no previous research has examined effects of chronic cannabis use on temporal order memory or most aspects of non-normative decision making, previous research has shown that chronic cannabis users demonstrate greater sunk cost propensity^21^ and a recent meta-analysis concluded that chronic cannabis use is associated with impaired prospective memory^33^. Future research employing an additional control group of sober non-users would permit for the examination of this possibility. Alternatively, our study was underpowered to detect small and medium sized effects, these tests (particularly the reminder prospective memory test which was scored in a binary manner and had a bit of a ceiling effect) may not have been sensitive enough to detect effects, and/or our sample of regular cannabis users may have developed tolerance to the impairing effects of cannabis, thereby diminishing our ability to detect group differences. Chronic cannabis users are known to become tolerant to the impairing effects of the drug^5–7^) as a result of down-regulation of CB1 receptors in the cortex following habitual cannabinoid exposure^34^. Nevertheless, as this is the first study to examine the acute effects of cannabis on prospective memory, temporal order memory, and non-normative decision making, further research with larger samples of less experienced cannabis users is needed before we can reach firm conclusions that acute cannabis intoxication has no impact on these aspects of cognition.

Comparisons of the effects of high-potency cannabis flower with and without CBD revealed that the THC + CBD flower group freely recalled significantly *fewer* pictures on the source memory test relative to the sober, THC flower, and concentrates groups. This contradicted our hypothesis, as well as previous research showing protective effects of CBD on memory^8,9^. It is possible that the CBD concentrations in the THC + CBD flower products used in this study were not substantial enough to produce the protective effects previously observed. While we aimed for higher CBD concentrations, they proved exceptionally difficult to find in high-potency flower. Nevertheless, even Morgan et al.^35^ were not able to replicate their finding that co-administration of CBD protects against the memory impairing effects of THC^9^. Indeed, there is contradictory evidence that CBD can potentiate effects of THC. Specifically, previous pre-clinical research has shown that administering CBD with or before THC can increase concentrations of THC, relative to administration of THC alone^11–15^. Human studies pertaining to pharmacokinetic interactions between THC and CBD are limited, but one study found that inhalation of cannabis containing 11% THC and 11% CBD resulted in higher plasma concentrations of THC than cannabis containing 11% THC and < 1% CBD, and in some cases, cannabis containing balanced THC:CBD ratios caused *greater* functional impairments than THC-dominant cannabis^10^. As such, products used by our THC + CBD flower group may have caused more potent effects than those used by the THC flower group, because CBD exacerbated effects of THC.

Results also failed to support our hypothesis that concentrates would exacerbate cognitive impairments. However, as previously discussed participants randomly assigned to use concentrates took significantly fewer puffs and subsequently self-reported the same level of intoxication as individuals randomly assigned to inhale flower. Once again, these important and novel findings suggest that all three cannabis-using groups self-titrated to achieve comparable levels of intoxication and impairment. Thus, it is possible that the flower and concentrates groups did not differ significantly in the overall amount of THC they ingested, despite differences in product potency. However, a recent study revealed dramatically higher blood concentrations of THC and its primary metabolite in individuals who had recently used a concentrate relative to those who had inhaled flower^36^. Nevertheless, that study also failed to detect greater cognitive impairments in concentrate users than flower users^36^. As this is only the second study to compare the cognitive effects of cannabis flower to cannabis concentrates in humans, additional research is urgently needed.

### Limitations and strengths

The primary limitation of this field experiment was the inability to include a placebo control group. While a sober control group was used, the lack of a placebo control group precludes the ability to rule out drug expectancy effects. However, it is notoriously difficult to effectively use placebos in acute cannabis studies because participants are often aware of the absence of feelings of intoxication and expectancy effects are an inherent component of real-life cannabis use, and therefore contribute to its real-world effects. Additionally, DEA restrictions prevented us from objectively verify the THC and CBD concentrations in the products used and there is evidence that the cannabinoid content provided on labels of cannabis products are not always accurate or consistent^37^. Another potential limitation was that participants vaped cannabis concentrates using vape pens and smoked cannabis flower in joint form as these are the methods by which these products are most commonly inhaled. Given vaping flower has been shown to increase plasma THC levels and exacerbate cognitive impairments relative to smoking flower^38^ future research should manipulate vaping/smoking in a more balanced manner. Finally, the study was exploratory in nature and was underpowered to detect small and medium sized effects. Additional studies with larger samples are required to determine the true absence of effects of high-potency cannabis products on prospective memory, temporal order memory, and non-normative decision making.

The primary strength of this study is the use of a novel methodology with high ecological validity. Context-specific effects of many drugs have been established^39^ and the experience of using cannabis in a laboratory setting likely differs from that of using it at home (e.g., the lab environment may be more anxiogenic). By testing participants in their homes, we were able to more accurately capture their natural experience of intoxication and the resulting effects on everyday life cognition. Moreover, despite recent trends toward the legalization of cannabis in numerous states, the U.S. federal government continues to classify cannabis as a Schedule I illicit drug^2^ and bars researchers’ access to the high-potency cannabis products popular in recreational dispensaries^1^. This has created an urgent need to develop novel methodologies to examine acute effects of cannabis on humans. By allowing participants to purchase their own cannabis using their own funds and testing them remotely while they use their own products in their own homes, this methodology effectively bypasses federal restrictions on the administration of cannabis for research purposes, and provides an ethical, safe, inexpensive, practical and discrete method of testing acute effects of cannabis on humans.
